# Refracture after surgical management of pediatric diaphyseal forearm fractures: a retrospective analysis

**DOI:** 10.3389/fped.2026.1785495

**Published:** 2026-05-29

**Authors:** Chen Zhang, Zhenkun Gu, Jianglong Xu, Zilong Huang, Dechao Wu, Jiansheng Wang, Guibing Fu

**Affiliations:** Department of Orthopedics, Shenzhen Children’s Hospital, Shenzhen, China

**Keywords:** child, diaphyseal forearm fracture, pediatric fracture, refracture, surgical management

## Abstract

**Purpose:**

Pediatric diaphyseal forearm fractures are common, and refracture represents a significant postoperative complication. This study aimed to investigate risk factors for refracture following pediatric diaphyseal forearm fractures and to delineate the clinical characteristics and trends of these refractures.

**Methods:**

A retrospective analysis was conducted on the medical records of 1,593 patients with diaphyseal forearm fractures treated at our hospital between January 1, 2019, and January 1, 2024. Data collection included demographics, fracture characteristics, and surgical details. Patients were divided into refracture and non-refracture groups. Univariate analysis was performed to explore factors associated with refracture. Clinical features of refracture, including injury mechanisms and timing, were also described.

**Results:**

Among 1,593 children with diaphyseal forearm fractures, 73 cases (4.58%) experienced refracture. The median time to refracture was 135 days (range: 35–362 days), with 47.95% occurring between 3 and 6 months post-initial fracture. Routine daily activities were the cause of injury in 64.38% of refracture cases. Univariate analysis revealed significant differences between the refracture and non-refracture groups regarding midshaft fracture location (*P* = 0.01), shorter initial immobilization (*P* = 0.04), and the presence of intramedullary fixation tip exposure (*P* = 0.02). The two groups showed no significant differences in demographic characteristics, including age, gender, body weight, and height, or in initial fracture characteristics or surgical methods.

**Conclusion:**

The incidence of diaphyseal forearm refractures in our cohort was 4.58%, with the highest risk occurring between 3 and 6 months post-fracture—often related to daily activities. Variables associated with refracture in univariate analysis included midshaft fracture location, exposed intramedullary fixation tips, and shorter initial immobilization. Clinicians should consider burying intramedullary fixation tips, and extending postoperative protection with bracing after cast removal.

## 1 Introduction

Diaphyseal forearm fractures (DFF) rank among one of the most frequent injuries in pediatric populations (1–4). While most heal without significant long-term consequences, the incidence of diaphyseal forearm refractures (DFR) has increased over recent decades (5, 6). Literature reports revealed a broad spectrum of DFR rates, spanning 0.9% to 8% (2, 7, 8). Refractures carry the risk of consequences, including malunion (improper healing), nonunion (failure to heal), the need for additional surgical procedures, prolonged recovery times, and lasting impairment of forearm strength and rotation (8). Despite their substantial clinical impact, consensus on the risk factors predisposing children to DFR remains poorly defined.

Existing literature highlights several risk factors for DFR in children, encompassing patient demographics, initial fracture characteristics, and treatment approaches. Notably, greenstick fractures with residual angulation are associated with a substantially increased risk of refracture, as the intact cortex may impair the healing process and result in incomplete consolidation (9). In addition, midshaft forearm fractures are associated with a higher refracture risk compared to other locations (5, 10). While strategies to reduce DFR risk—such as extended immobilization, post-cast bracing (though evidence conflicts), optimizing surgical fixation, and prolonged activity restriction—have been explored, their effectiveness often lacks robust validation, and high-level evidence is scarce (2, 4, 11).

This study aims to investigate the risk factors associated with DFR in pediatric patients, characterize their clinical features, and identify emerging trends. The findings seek to inform improvements in treatment strategies, ultimately aiming to reduce the occurrence of DFR in children.

## 2 Methods

**Figure 1:**
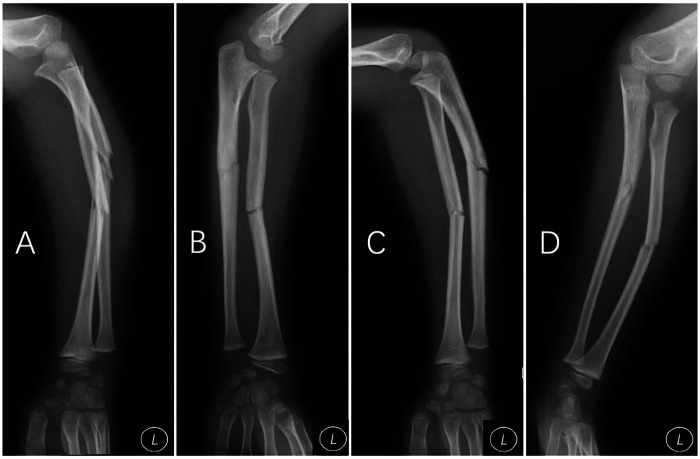
A 4-year-old male's primary forearm fracture (x-rays **A, B**) and refracture (x-rays **C, D**) occurred at the same site, as confirmed by anteroposterior and lateral comparisons.

This retrospective, single-center case series reviewed patients treated between January 2019 and January 2024. DFF was characterized as a fracture located between the proximal and distal metaphysis of the radius and/or ulna (12). Refracture was defined as a subsequent fracture at the same anatomical site within one year of the initial injury. Primary and subsequent fracture radiographs were compared to confirm the location (Figure 1). We included medical records of all patients ≤18 years diagnosed with DFF at our center. Patients were excluded for: (1) pathological fractures, (2) follow-up less than one year. Data collection of patient characteristics included age, sex, height, weight, injury mechanism, and the time interval to refracture. Fracture sites were assessed using anteroposterior and lateral radiographs.

All surgeries utilized general or regional anesthesia. Closed reduction was the initial approach, unsuccessful attempts were followed by open reduction and intramedullary fixation using Kirschner wires or intramedullary nails, followed by external fixation with a cast. Surgical data included the specific procedure performed and whether the implant ends were exposed. The timing of internal implant removal was determined by the physician based on radiographic assessment of bone callus, physical examination of the affected limb, and the presence of any surgical site infection. Cast immobilization was maintained during this period, and the final cast removal time was also recorded.

The Kolmogorov–Smirnov (KS) test was used to assess whether continuous variables followed a normal distribution. Continuous variables with a normal distribution were reported as mean ± standard deviation (SD) and compared using the independent Student's t-test. Non-normally distributed variables were reported as median (minimum-maximum) and compared using the Wilcoxon rank-sum test. Categorical variables were analyzed using the *χ*^2^ test. A Kaplan–Meier analysis with 95% confidence intervals (CIs) was used descriptively to illustrate the temporal distribution and cumulative occurrence of refracture after the primary injury. Statistical analyses were conducted using R (Version 4.4.2; R Core Team, 2024). Statistical significance was set at *p* < 0.05.

## 3 Results

The study cohort comprised 1,593 patients, including 662 females and 931 males. Among them, 73 patients experienced refracture, accounting for 4.58% of the total sample. Demographic characteristics (age, gender, body weight, and height) and initial fracture characteristics showed no significant differences between the two groups (Table 1).

**Table 1:** Comparison between patients with and without refractures.

Characteristics	No-Refractured (*n* = 1520)	Refractured (*n* = 73)	*p*
Age (year)	7.1 ± 2.6	8.2 ± 2.3	0.10
Body weight (kg)	27.5 (15–55)	29.5 (20–63)	0.36
Height (cm)	137 (96–170)	126 (91–166)	0.22
Gender			0.50
male	886	46	
female	634	27	
Open fracture			0.90
Yes	57	2	
No	1463	71	
Fracture location			0.01
Upper third	233	7	
Middle third	769	50	
Low third	518	16	
Surgical Approach			0.15
Open reduction	459	29	
Closed reduction	1061	44	
Intramedullary fixation time (days)			0.11
Kirschner wires	42 (32 -64)	44 (30–87)	
Intramedullary nails	130 (62–207)	141 (85–247)	
Immobilization time (days)	45 (36–70)	41 (33–75)	0.04
Intramedullary fixation tips			0.02
Exposed	944	55	
Buried	576	18	

Among all patients, 819 cases involved fractures in the middle third of the radial and ulnar shafts. The incidence of middle-third fractures was significantly higher in the refracture group compared to the non-refracture group (75.34% vs. 62.11%, *p* = 0.02). No statistically significant difference was observed between the refracture and non-refracture groups in the choice of closed vs. open reduction. However, the proportion of patients with exposed intramedullary fixation tips was higher in the refracture group than in the non-refracture group (75.34% vs. 62.11%, *p* = 0.02). The immobilization duration was 41 days (range: 33–75 days) in the refracture group and 45 days (range: 36–70 days) days in the non-refracture group. A statistically significant difference in immobilization duration was observed between the two groups, although the absolute difference was small (Table 1).

Characteristics of the 73 refracture patients are detailed in Table 2. The median time to refracture was 135 days (range: 35–362 days). Falls were the primary mechanism mainly from ground level, followed by sports injuries, mostly during recreational activities.

**Table 2:** Refracture characteristics.

Characteristics	Value
Total number (n)	73
Days to first refracture (days)	135 (35–362)
Mechanism of injury, (n/%)	
Fall	51 (69.86%)
ground-level fall*	47 (64.38%)
fall from height	4 (5.48%)
Sports	22 (30.14%)
related recreational activities**	16 (21.92%)
related competitive sports***	6 (8.22%)

* e.g., walking or playing.

** e.g., bicycle, skating, skateboard, and slide.

*** e.g., basketball or football.

**Figure 2:**
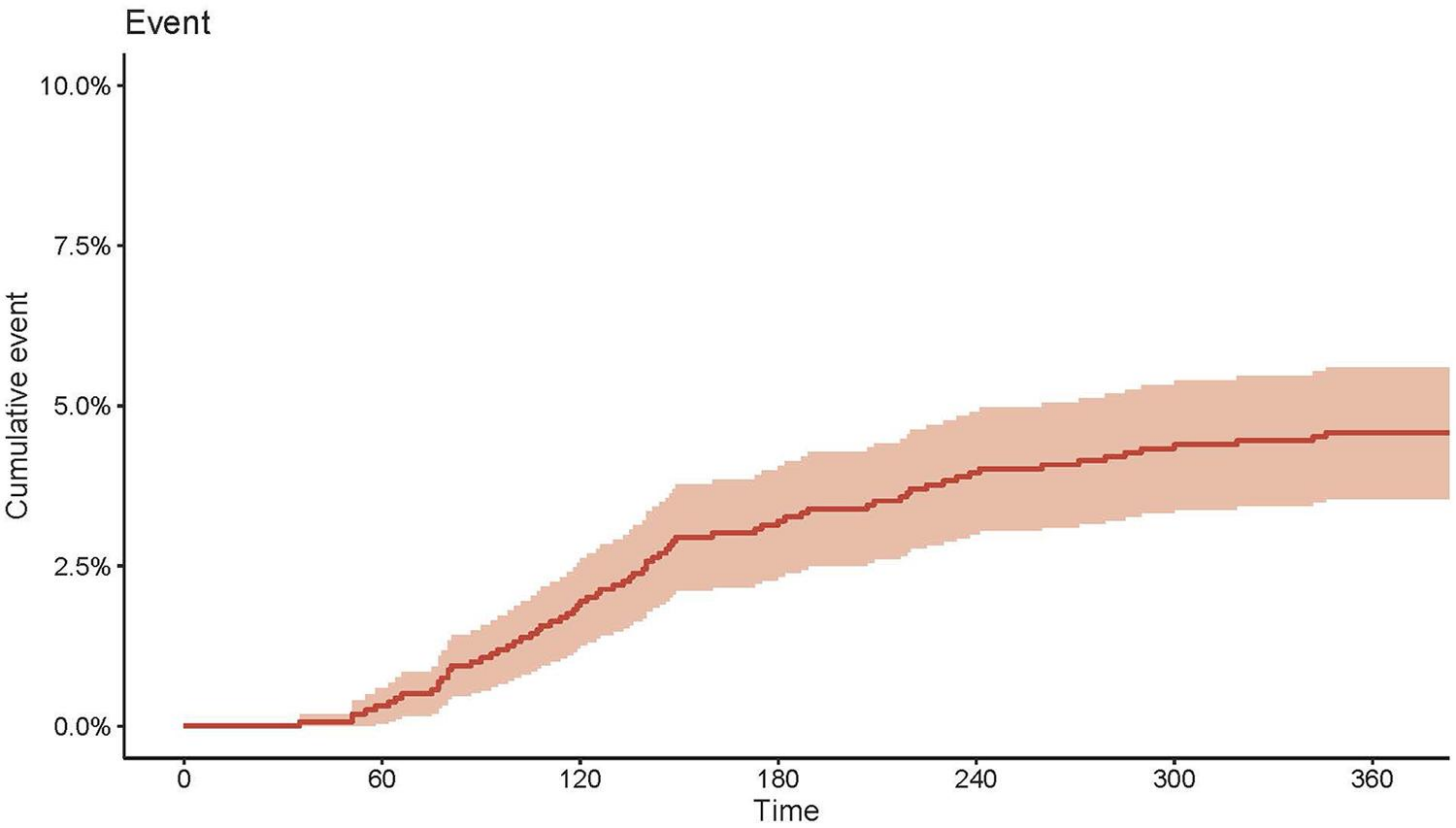
Kaplan–meier curve showing the temporal distribution and cumulative occurrence of refracture after the primary fracture.

The Kaplan–Meier curve (Figure 2) was used descriptively to illustrate the timing and cumulative occurrence of refracture after the initial injury. Refractures occurred most frequently between 3 and 6 months post-injury, accounting for 47.95% of all events.

## 4 Discussion

Refracture following operative treatment of pediatric DFF remains a significant clinical concern, potentially leading to prolonged morbidity (7). While overall rates are documented, a clearer understanding of specific risk factors and optimal post-operative management strategies is crucial for improving outcomes. This study aimed to identify key risk factors associated with refracture in our patient cohort and contextualize these findings within the existing literature to refine prevention strategies.

In our study, the overall refracture rate was approximately 4.58%, which aligns with previously reported ranges (0.9%–8%) (2, 7, 8). Univariate analysis showed statistically significant differences between the two groups in fracture location, immobilization duration, and exposure of intramedullary fixation tips. However, the difference in immobilization duration was small, and its clinical significance should be interpreted with caution. Notably, the highest incidence of refracture occurred between three and six months after the initial fracture, predominantly resulting from low-energy injuries sustained during routine daily activities. It should be noted that the definition of refracture varies in the literature. Some authors distinguish refractures related to incomplete recovery after minor trauma from new fractures caused by sufficient trauma at the same site (9). In our study, refracture was defined as a repeat fracture occurring at the same location within one year of the primary injury, consistent with previously published criteria (13). This difference in definition should be considered when comparing results across studies.

Consistent with previous reports, our findings confirm that midshaft DFF carry a higher refracture risk compared to proximal or distal third fractures (2, 5, 13, 14). This vulnerability is likely attributable to increased muscular deforming forces and greater demands on callus formation in the diaphyseal region, potentially slowing the healing process (4, 14, 15).

Our finding that exposed intramedullary fixation tips increase the risk of refracture corroborates previous research (10, 16, 17). Prévot and colleagues recommended burying the ends of the intramedullary nails and retaining the implants for 6–9 months in order to prevent refracture (18). Intramedullary fixation not only protects the fracture site but also serves as a reminder to patients that treatment is ongoing (10). However, exposed tips may cause complications such as irritation or infection, often prompting premature removal before robust healing is achieved (17). Early removal eliminates both the mechanical protection and the behavioral reminder provided by the implant (10). Although some debate exists regarding whether early vs. late removal of buried tips affects complication rates, our data support burying intramedullary fixation tips to enable longer implant retention, thereby promoting uninterrupted healing and reducing refracture risk (19). However, the decision to bury the nail tips must be balanced against the potential challenges during implant removal. Retrieval of buried implants often requires hospital admission, more extensive surgical dissection, larger incisions, and possibly additional bone removal, and may be associated with increased medical costs. Therefore, the recommendation to bury nails should be individualized, weighing the potential reduction in refracture risk against the increased complexity of future hardware removal surgery.

Some studies have suggested that insufficient immobilization may be associated with an increased risk of refracture (2, 11, 14). In our cohort, although immobilization duration differed statistically between the two groups, the absolute difference was small (median, 41 vs. 45 days), and therefore its clinical significance should be interpreted cautiously. Previous literature recommends 6–8 weeks of cast immobilization, with return to activity at 3 months when the fracture line tends to disappear (2, 15, 20). Although the treatment protocols in our study conformed to these established guidelines, refractures still occurred. Moreover, we observed that the peak refracture incidence occurs between 3 and 6 months, often during daily activities, raising questions about the sufficiency of current standards. These observations highlight a delicate balance and suggest that the need for prolonged protection may be underestimated, especially as patients resume activity. It should be noted that some previous studies have not advocated additional cast immobilization with following flexible intramedullary nailing for forearm fractures (21). However, owing to regional variations, differences in patient activity levels, or subtle technical aspects of surgical management, our institutional protocol includes postoperative cast immobilization. Our data also demonstrate that the use of cast for additional external protection helps reduce the risk of refracture, thereby offering reassurance to clinical centers that utilize the same treatment approach.

In this study, we acknowledge several limitations inherent to its retrospective and single-center design. First, as a retrospective analysis, there is the potential for selection bias. In addition, activity-related variables, such as sports participation and baseline activity level, were not systematically recorded across the entire cohort and therefore could not be included in the analysis, although they may have influenced refracture risk. Additionally, recall bias could have affected the accuracy of the clinical data collected, especially regarding patient histories and follow-up outcomes. Moreover, the lack of randomization means that our results may not be applicable to all clinical settings, as treatment protocols may differ across centers. Finally, the single-center nature of the study further limits the external validity of the findings, as practices at our institution may not be reflective of those at other medical centers. These limitations highlight the need for prospective, multicenter studies to validate the findings and reduce the potential for bias.

In conclusion, the incidence of DFR in our study was approximately 4.58%. Our findings suggest that midshaft fracture location, exposed intramedullary fixation tips, shorter initial immobilization periods, and the critical 3–6-month post-injury period (associated with daily activity injuries) may be associated with pediatric DFR risk. These findings suggest that current immobilization protocols may be insufficient for all patients. To reduce refracture risk, clinicians should consider burying intramedullary fixation tips, extending postoperative protection with bracing after cast removal.

## Data Availability

The raw data supporting the conclusions of this article will be made available by the authors, without undue reservation.
